# Quality of life and psychometric evaluation of patients diagnosed with irritable bowel syndrome: an observational cohort study

**DOI:** 10.1590/1516-3180.2019.0527.R1.16042020

**Published:** 2020-06-15

**Authors:** Alp Yildiz, Emre Kizil, Aybala Yildiz

**Affiliations:** I MD. General Surgeon, Department of General Surgery, Yenimahalle Training and Research Hospital, Yildirim Beyazıt University, Ankara, Turkey.; II MD. Psychiatrist, Private Emre Kizil Psychiatry Clinic, Cankaya, Ankara, Turkey.; III MD, PhD. Associate Professor, Department of General Surgery, Yenimahalle Training and Research Hospital, Yildirim Beyazıt University, Ankara, Turkey.

**Keywords:** Anxiety, Depression, Quality of life, Irritable bowel syndrome, Simethicone combination, Otilonium bromide, Psychosomatic disorders

## Abstract

**BACKGROUND:**

Very few data are available for evaluating health-related quality of life among people with irritable bowel syndrome (IBS) and even fewer data are available in relation to anxiety and depression status among these patients.

**OBJECTIVES:**

To evaluate the quality of life, anxiety and depression status of patients with IBS.

**DESIGN AND SETTING:**

Observational cohort study conducted in a tertiary-care university hospital.

**METHODS:**

Patients who had recently been diagnosed with IBS and who had been followed up for IBS-specific treatment for at least three months were included. A quality of life (QoL) survey, the Beck Anxiety Index (BAI) and the Hamilton Depression Index (HAM-D) were applied to the patients.

**RESULTS:**

In total, 274 patients with IBS were included in the study cohort. These patients presented very high baseline scores for anxiety and depression, and very poor QoL results.

**CONCLUSION:**

Our study showed that IBS had a very high impact on these patients, regarding their anxiety and depression levels, alongside very poor results relating to quality of life.

## INTRODUCTION

Irritable bowel syndrome (IBS) is a functional bowel disorder that gives rise to deterioration of quality of life (QoL). The global prevalence of IBS has been estimated to be 11.2%.^1^ IBS is the second most common cause of productivity loss in the United States.^1^^,^^2^ The pathogenesis of IBS is multifactorial, and the diagnosis is made based upon the presenting symptoms and on ruling out organic diseases.^3^^,^^4^^,^^5^

## OBJECTIVE

The main question and aim of this study was to demonstrate the psychometric status of patients with IBS as a functional chronic disease. Hence, the aim was therefore to evaluate the QoL and psychological profile of patients with concurrent IBS within current medical practice.

## METHODS

This observational cohort study was conducted retrospectively between March 2016 and January 2019. The subjects were adult patients who had been referred to the general surgery clinic with gastrointestinal symptoms and were diagnosed and treated as IBS cases, in accordance with the Rome-IV criteria.

The patients were asked to complete a QoL survey and the questionnaires of the Beck Anxiety Index (BAI) and Hamilton Depression Index (HAM-D) before their IBS treatment and three months afterwards. The aim of this was to monitor the patients’ psychological condition. The severity of IBS was then re-evaluated using the Rome IV criteria, with the aim of documenting any occurrence of remission following the treatment regimen, at the end of the three-month period.

For this study, the files of 614 IBS patients were evaluated. Out of these 614 patients, 274 who completed all questionnaires both before and three months after the treatment were enrolled in this retrospective study.

All the patients were assessed by means of upper gastrointestinal endoscopy, rectosigmoidoscopy, routine laboratory tests and abdominal ultrasonography. Patients with unstable systemic diseases or previous psychiatric disorders were excluded. Patients who had been taking proton pump inhibitors for at least three months for other reasons (because of chronic use of anti-inflammatory drugs due to orthopedic conditions or chronic use of acetyl salicylic acid due to neurological conditions, etc.) were included the study. However, patients with late onset of proton pump inhibitors (less than three months) were excluded from the study.

Newly diagnosed IBS patients were treated with a combination product consisting of otilonium bromide (40 mg) and simethicone (80 mg), administered orally three times per day at mealtimes.

We had already recorded data on treatment outcomes for supportive reasons, and so we report these data here. Nonetheless, because the present study had a retrospective design, these data were insufficient for evaluating treatment efficacy.

### Statistics

The SPSS 25.0 software (IBM Corporation, Armonk, New York, United States) was used in the analysis on the variables. The variables were analyzed at a 95% confidence level, and a P-value of less than 0.05 was considered significant. The Mardia (Doornik and Hansen omnibus) test was used to test whether the data conformed to a multivariate normal distribution, while the homogeneity of variance was evaluated using Box’s M test. The Mann-Whitney U test was used with Monte Carlo scores to compare before-and-after differences in the measurements of fibromyalgia symptom severity scores and in the BAI, quality-of-life and HAM-D scales, in relation to each other, according to whether individuals achieved complete recovery or did not.

The Wilcoxon signed-rank test was used with Monte Carlo results to compare before-and-after measurements of fibromyalgia symptom severity scores and the BAI, quality-of-life and HAM-D scales. The marginal homogeneity test was used with the Monte Carlo simulation method to compare before-and-after measurements of the classified BAI and HAM-D scores. The Pearson chi-square test was used with the exact and Monte Carlo simulation methods, and the Fisher-Freeman-Halton exact test was used with the Monte Carlo simulation method, to compare the BAI and HAM-D scales. The subjects were classified according to whether complete recovery had been achieved or not. Column ratios were compared and expressed in accordance with the Benjamini-Hochberg corrected P-value results. Quantitative variables were expressed in the tables as median (minimum/maximum) and categorical variables were shown as n (%). This retrospective study received local ethics committee approval under the registration number VDH.271010/1124, approved on October 27, 2010.

## RESULTS

The present study was carried out with a total cohort size of 274 patients with irritable bowel syndrome. Among these patients, 219 were female and 55 were male. The patients’ mean age was 44.1 ± 5.5 years; the oldest was 84 and the youngest was 18 years of age. Out of the 274 patients included in the study, 99 (36.1%) did not have complete recovery, while 175 (63.9%) had complete recovery from irritable bowel syndrome (Table 1).

**Table 1.** Psychometric score results from the two groups

	Total	Complete recovery (absent)	Complete recovery **(present)**	P-value for complete recovery
	(n = 274)	(n = 99)	(n = 175)	
	Median (minimum/maximum)	Median (minimum/maximum)	Median (minimum/maximum)	
Beck Anxiety Inventory				
Before	10 (1/58)	5 (1/16)	22 (1/58)	< 0.001^U^
After	6 (1/33)	6 (1/9)	8 (1/33)	0.001^U^
Difference (before-to-after)	-4 (-44/8)	0 (-12/8)	-7 (-44/7)	< 0.001^U^
P-value for before-to-after^W^	< 0.001	0.364	< 0.001	
Quality-of-life scale				
Before	3 (14/58)	43 (14/ 8)	43 (14/58)	0.992^U^
After	88 (51/104)	88 (51/104)	88 (51/104)	0.665^U^
Difference (before-to-after-)	45 (2/90)	43 (2/90)	46 (3/82)	0.511^U^
P-value for before-to-after^W^	< 0.001	< 0.001	< 0.001	
Hamilton Depression Rating Scale (HAM-D)				
Before	6 (5/22)	5.5 (5/6)	7.5 (5/22)	< 0.001^U^
After	2 (1/21)	2 (1/6)	4 (1/21)	< 0.001^U^
Difference (before-to-after)	-4 (-17/4)	-4 (-5/0)	-4 (-17/4)	0.971^U^
P-value for before-to-after^W^	< 0.001	< 0.001	< 0.001	

^U^Mann-Whitney U test (Monte Carlo); ^w^Wilcoxon signed-rank test (Monte Carlo).

There were no statistically significant differences in the quality-of-life scores before the pharmacotherapy or after the pharmacotherapy or in the before-to-after difference in scores, between patients who achieved complete recovery (P = 0.781, 0.304 and 0.395, respectively) and those who did not (P = 0.992, 0.665 and 0.511, respectively), i.e. P > 0.05 for all comparisons. However, on the HAM-D scale, there was no significant difference between those who had complete recovery and those who did not, in terms of before-to-after difference (P = 0.971).

The median values on the Beck Anxiety Inventory (BAI) scale before pharmacotherapy and after pharmacotherapy and the before-to-after difference of those who achieved complete recovery (median (minimum/maximum)) were 9 (7/12), 3 (1/8) and -6 (-11/-1), respectively. These were statistically significantly higher than the median values of those who did not reach complete recovery (median (minimum/maximum)), which were 9 (7/12), 3 (1/9) and -6 (-11/1), respectively (P < 0.001, 0.001 and < 0.001, respectively).

The median values on the HAM-D scale before pharmacotherapy and after pharmacotherapy of those who had complete recovery (median (minimum/maximum) were 7.5 (5/22) and 4 (1/21), respectively. These were statistically significantly higher than the median values of those who did not reach complete recovery (median (minimum/maximum)), which were 5.5 (5/6) and 2 (1/6), respectively (P < 0.001).

Among all the patients, and among those who had complete recovery, there were statistically significant decreases in the median BAI and HAM-D scores after the pharmacotherapy (all P-values < 0.001), while the median values on the quality of life scale increased after the pharmacotherapy, compared with the values before the pharmacotherapy (P < 0.001).

However, among the patients without complete recovery, there were statistically significant decreases in median HAM-D scores after the pharmacotherapy, compared with before it (all P-values < 0.001), while the median values on the quality of life scale increased after the pharmacotherapy, compared with before it (P < 0.001). There was no statistically significant difference in relation to the BAI scale (P = 0.364) (Table 1).

There were statistically significant differences in the distribution ratios of the classified BAI and HAM-D scales from before to after the pharmacotherapy, in a comparison according to the complete recovery situation (all P-values < 0.001).

On the BAI scale, before the pharmacotherapy, presence of a low anxiety ratio among the patients without complete recovery (100%) was greater than among those with complete recovery (48%). On the other hand, presence of medium anxiety ratios (33.1%) and high anxiety ratios (18.9%) was greater among patients with complete recovery (P < 0.05). Presence of a low anxiety ratio (100%) after the pharmacotherapy was greater among the patients without complete recovery than among those with complete recovery (84.6%), while presence of a medium anxiety ratio was greater among those with complete recovery (15.4%) (P < 0.05).

Among all the patients, and among those with complete recovery, there were decreases in the numbers of patients presenting medium and high anxiety levels both before and after the pharmacotherapy, while there was an increase in the number of patients with low anxiety (all P-values < 0.001). However, among those with complete recovery, there was no significant difference (P = 0.999)

Among those without complete recovery (100%), the rate of incidence of normal HAM-D scores before the pharmacotherapy was higher than among those without complete recovery (51.4%). However, mild (17.7%), medium (33.1%), severe (21.7%) and very severe (9.1%) depression levels were higher among those with complete recovery (P < 0.05). The rate of incidence of normal scores among patients without complete recovery after the pharmacotherapy (100%) was higher than among those with complete recovery (67.4%), while mild (24%) and medium (6.3%) depression ratios were higher among those with complete recovery (P < 0.05).

Among all the patients, and among those with complete recovery, there were decreases in the depression ratios after the pharmacotherapy among the patients with mild, medium, severe and very severe depression, compared with their ratios before the pharmacotherapy, while there was an increase in the rate among normal patients (all P-values < 0.001). However, there was no significant change among those with complete recovery (P = 0.999) (Table 2).

**Table 2.** Detailed psychometric values for the patients, according to subgroups

	Total	Complete recovery (absent)	Complete recovery (present)	P-value for complete recovery
	(n = 274)	(n = 99)	(n = 175)	
	n (%)	n (%)	n (%)	
Beck Anxiety Inventory				
Before				
Low	183 (66.8)	99 (100)^B^	84 (48)	< 0.001^PM^
Moderate	58 (21.2)	0 (0)	58 (33.1)^A^	
Severe	33 (12)	0 (0)	33 (18.9)^A^	
After				
Low	247 (90.1)	99 (100)^B^	148 (84.6)	< 0.001^PE^
Moderate	27 (9.9)	0 (0)	27 (15.4)^A^	
Severe	0 (0)	0 (0)	0 (0)	
P-value for before-to-after^MH^	< 0.001	0.999	< 0.001	
Hamilton Depression Rating Scale (HAM-D)				
Before				
Normal	189 (69)	99 (100)^B^	90 (51.4)	< 0.001^PM^
Mild	31 (11.3)	0 (0)	31 (17.7)^A^	
Moderate	38 (13.9)	0 (0)	38 (21.7)^A^	
Severe	16 (5.8)	0 (0)	16 (9.1)^A^	
Very severe	0 (0)	0 (0)	0 (0)	
After				
Normal	217 (79.2)	99 (100)^B^	118 (67.4)	< 0.001^FF^
Mild	42 (15.3)	0 (0)	42 (24)^A^	
Moderate	11 (4)	0 (0)	11 (6.3)^A^	
Severe	4 (1.5)	0 (0)	4 (2.3)	
Very severe	0 (0)	0 (0)	0 (0)	
P-value for before-to-after^MH^	< 0.001	0.999	< 0.001	

^P^Pearson chi-square test (Exact, Monte Carlo), ^FF^Fisher-Freeman-Halton test (Monte Carlo), ^MH^marginal homogeneity test (Monte Carlo), ^A^expressed significance for the group without complete recovery, ^B^expressed significance for the group with complete recovery.

## DISCUSSION

The relationship between functional somatic syndromes and IBS remains unclear, but co-occurrence of IBS and fibromyalgia has been reported to be high in the literature. In a study investigating separate groups of IBS patients, 65% of IBS patients were found to suffer from other psychosomatic disorders and 70% of psychosomatic disorders patients had IBS symptoms,^6^^,^^7^ which suggests that these conditions have a common etiology. General anxiety disorder, depression and/or depressive symptoms have also been found to be consistently higher in IBS patients than in healthy controls.^8^^,^^9^^,^^10^

It is known that bowel habits change, and IBS is seen frequently in the course of major depressive disorder (MDD). Tollefson et al.^11^ reported that the IBS criteria were met in 30% of patients with MDD, whereas the IBS rate in the psychiatrically healthy control group remained at 11%. Masand et al.^12^ found that the incidence of IBS in MDD patients was similar to that of Tollefson (27%), whereas the IBS rate in the control group remained at 3%. In another study, the incidence of IBS in patients with major depressive disorder (double depression) that developed on the basis of dysthymia reached 58%, while the rate in the control group was again limited to 3%.^11^^,^^12^

Mayer et al.^13^ reported that IBS was accompanied by anxiety in the early period and depression in the late period. Despite the epidemiological data available, they suggested that IBS, which is seen with depression and anxiety disorders, leads patients to seek help more frequently and that the rates determined in studies may be misleading because of these characteristics. In fact, they claimed that more than 50% of patients seeking treatment for IBS also had depression and anxiety disorders.^13^

The following questions need to be considered: Why does IBS not develop in anyone who has inflammation or visceral hypersensitivity in the colon mucosa? and, Why is depression not seen in all patients with IBS? The possible answers to these questions include individual dietary differences, genetic causes, frequency of bowel infections and susceptibility to anxiety and depression.^11^^,^^12^^,^^13^^,^^14^^,^^15^ If the pathophysiological link between IBS and MDD is summarized, a pathway extending from the colon mucosa to the anterior cingulate cortex may be suggested.^14^ Mucosal inflammation triggered by stress or infections increases cytokine levels and visceral sensory conduction mediated by neurokinins.^14^^,^^15^

Therefore, IBS treatment is expected to be effective for treating comorbid depression. The fact that the accompanying depression is more severe in IBS patients, which leads to higher levels of seeking help and appointments with doctors, makes the importance of our results greater. From this point of view, although IBS treatment can be thought to be helpful for these patients, our results regarding high anxiety and depression levels suggest that it would be very useful to take into account the results from this study. It seems that use of psychotherapy methods can also be beneficial.^11^^,^^14^^,^^16^^,^^17^

This study was designed as a real-world observational study and did not apply randomization or blind administration of medication. A high number of patients were lost to follow-up or did not complete the surveys correctly, and therefore were excluded from the analysis group. These are the weaknesses of our study. Moreover, although we reported the psychometric results subsequent to treatment, these results from a retrospective study design are insufficient for any kind of evaluation of treatment efficacy.

Our primary aim with this study was to demonstrate the levels of anxiety and depression, which were higher than expected, and the effect of irritable bowel syndrome on the patients’ quality of life. Therefore, we did not discuss the efficacy of the medication that was used in this study. In addition, we think that further prospective randomized controlled trials evaluating the effect of medication on improvement of quality of life and diminution of anxiety and depression levels are needed.

## CONCLUSION

This study showed that the great majority of IBS patients have very high levels of anxiety and depression and very poor levels of quality of life. In the light of our study, it can be suggested that in considering IBS treatment, both the physiological and the psychological aspects should be addressed, so as to maintain optimal results and enhance the quality of life of individuals with IBS.
